# The Role of the Adipokine Leptin in Immune Cell Function in Health and Disease

**DOI:** 10.3389/fimmu.2020.622468

**Published:** 2021-01-29

**Authors:** Kaitlin Kiernan, Nancie J. MacIver

**Affiliations:** ^1^Department of Immunology, Duke University School of Medicine, Durham, NC, United States; ^2^Department of Pediatrics, Duke University School of Medicine, Durham, NC, United States; ^3^Department of Pharmacology and Cancer Biology, Duke University School of Medicine, Durham, NC, United States

**Keywords:** leptin, obesity, inflammation, adaptive immunity, adipose tissue

## Abstract

Leptin is a critical mediator of the immune response to changes in overall nutrition. Leptin is produced by adipocytes in proportion to adipose tissue mass and is therefore increased in obesity. Despite having a well-described role in regulating systemic metabolism and appetite, leptin displays pleiotropic actions, and it is now clear that leptin has a key role in influencing immune cell function. Indeed, many immune cells have been shown to respond to leptin directly *via* the leptin receptor, resulting in a largely pro-inflammatory phenotype. Understanding the role of adipose-tissue derived mediators in inflammation is critical to determining the pathophysiology of multiple obesity-associated diseases, such as type 2 diabetes, autoimmune disease, and infection. This review, therefore, focuses on the latest data regarding the role of leptin in modulating inflammation.

## Introduction

Obesity is associated with a chronic, low-grade systemic inflammation that has been shown to promote the development of multiple disorders of health including type 2 diabetes, autoimmunity, nonalcoholic fatty liver disease, asthma, and cardiovascular disease (1, 2). This obesity-associated inflammation is characterized by increased circulating inflammatory cytokines such as tumor necrosis factor (TNF)and interleukin 6 (IL-6) as well as an increase in pro-inflammatory immune cells, particularly macrophages and lymphocytes (3–9).

The etiology of obesity-associated inflammation is complex. While many tissues demonstrate obesity-associated inflammation, adipose tissue is considered to be the central or key site of inflammation, responsible for driving systemic inflammation and disease (10, 11). Adipose tissue is altered in obesity, leading to increased adipocyte volume and lipid content. These alterations are associated with changes in adipose tissue-resident immune cells, characterized by an increase in immune cell number, particularly pro-inflammatory macrophages and lymphocytes (12–20). Inflammatory immune cells found within adipose tissue in obesity in turn promote adipocyte production of inflammatory molecules (21). Adipose tissue production of the pro-inflammatory hormone leptin, and the role of leptin in mediating obesity-associated inflammatory disease, is the subject of this review.

Leptin can be produced by multiple cells in the body, including immune cells, but is primarily produced by adipocytes in proportion to adipocyte mass, such that increasing adiposity leads to increased systemic concentrations of leptin (22, 23). Although leptin is produced in a diurnal manner (24), it is not a fast-acting signal or cytokine, but rather communicates stable nutritional status to the body as a whole. Leptin has a well-defined role as a metabolic mediator and communicator of nutritional status at the level of the hypothalamus where leptin receptors are highly expressed. Increased leptin signaling at the hypothalamus regulates appetite and leads to decreased nutrient intake and increased energy expenditure. Studies of leptin deficiency and fasting have demonstrated that leptin signaling is also required for normal reproductive hormone production, as well as thyroid hormone. Therefore, leptin plays a critical role in controlling energy homeostasis, metabolism, and neuroendocrine function. These functions of leptin have been thoroughly reviewed (25–27).

Over the last two decades, it has become apparent that leptin also has a critical role as an immune modulator. This was initially observed in individuals with rare mutations in leptin or the leptin receptor, who are obese from lack of leptin signaling at the hypothalamus, but were also found to have an increased risk of intracellular infections secondary to immune cell deficiencies (28). Leptin has subsequently been shown to act on several different immune cell types and can affect both immune cell development and function. Through that mechanism, increased systemic leptin levels in diet-induced obesity directly promote obesity-associated inflammation.

Leptin receptor is expressed by most cells of the immune system and many immune cells have been shown to be leptin responsive to varying degrees. In general, leptin receptor expression is important for hematopoietic cell development, immune cell proliferation and survival, and pro-inflammatory function (29, 30). In this review, we will characterize the effects of leptin on innate and adaptive immune cells, with a particular focus on CD4^+^ T cells, which are known to be highly leptin responsive, as summarized in **Table 1**. We will explore the mechanisms by which leptin is proposed to act on these cells, both through traditional signaling pathways and through altering cellular metabolism, much of which has been discovered in the mouse model. Finally, we will review the effects of leptin in human studies and identify the clinical relevance of this adipokine in the setting of both health and disease. Although leptin may have a role as a nutritional regulator of immunity in the setting of both under- and overnutrition, we will focus here on the effects of leptin on the immune system in the context of obesity.

**Table 1:** Distinct effects of leptin across immune cell types.

Immune cell	Leptin Effect
CD4^+^ T cells	Required for T cell development in the thymus (31–34) Increases proliferation of naïve T cells (35, 36) Promotes Th1 cytokine production (35) Promotes Th17 differentiation and cytokine production (34) Promotes increased glycolytic metabolism (31, 34)
B cells	Reduces apoptosis (37) Promotes cell cycle entry (37) Increases inflammatory cytokine production (38) Reduces class switching and IgG production (38)
Macrophages	Promotes bacterial clearance and phagocytosis (39, 40)
Monocytes	Increases TLR2 expression (41) Promotes inflammatory cytokine production (42)
Mast Cells	Promotes mast cell phenotype that drives inflammatory M1-like macrophage cell phenotype (43)
Dendritic cells	Reduces apoptosis by increasing expression of Bcl-2 and Bcl-xL (44) Promotes DC maturation and function (45) Increases inflammatory cytokine production (44)
Neutrophils	Inhibits apoptosis (46) Acts as chemoattractant (22, 47) Increases oxidative species production (48)
Basophils	Inhibits apoptosis (49, 50) Acts as chemoattractant, promotes trafficking toward other chemo attractants such as eotaxin (49) Increases IL-4 and IL-13 production (49)
Eosinophils	Inhibits apoptosis (49, 50) Acts as chemoattractant, promotes trafficking toward other chemo attractants such as eotaxin (51)
NK cells	Brief exposure promotes increased cytotoxicity (52) 18-h exposure increases IFN-γ and perforin production (52, 53) 72-h exposure inhibits IFN-γ and cytotoxicity (52)
ILCs	Promotes type-2 cytokine production (54)

## Adaptive Immune Cells

The effect of leptin on immune cells has been best studied in the context of adaptive immunity, particularly its effects on CD4^+^ T cells. Leptin has been shown to have a role in modulating T cell development, as well as T cell function and metabolism. Moreover, distinct functional CD4^+^ T cell subsets respond to leptin in different ways that reflect their function. CD8^+^ T cell and B cell responses to leptin have also been studied, but to a lesser extent.

### T Cells

Leptin plays an important role in T cell development. Leptin deficiency has been shown to result in thymic atrophy and decreased circulating T cell numbers (31, 33, 34). Interestingly, leptin receptor has been found to be expressed on double negative, double positive and CD4 single positive thymocyte subsets, but not on CD8 single positive thymocytes (32). Moreover, leptin treatment rescued CD4^+^ T cell development in leptin mutant (*ob/ob*) mice, but did not rescue CD8^+^ T cell development (32). Together this suggests that leptin is required for early T cell development and for later development of CD4^+^ T cells, but not CD8^+^ T cells.

**Figure 1:**
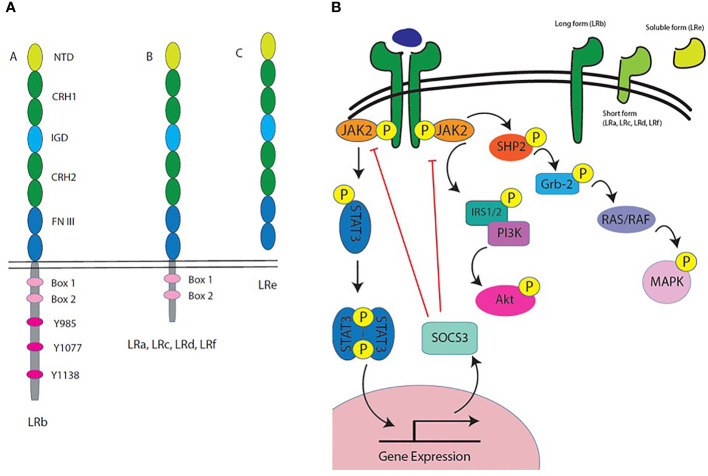
Leptin receptor isoforms and intracellular signaling. **(A)** Leptin receptor is composed of an extracellular domain, a transmembrane domain, and a cytoplasmic domain. All variants of the leptin receptor include the extracellular domain. The extracellular domain is composed of several protein motifs: the N terminal domain (NTD), two cytokine receptor homology (CRH) domains that make up the leptin binding site, an immunoglobulin-like domain (IGD), and two fibronectin type 3 (FN III) domains. The cytoplasmic domain of leptin receptor varies between isoforms. LRb, the long form receptor, includes two box domains and several tyrosine residues important for leptin receptor signaling. The other leptin receptor variants are labeled LRa, LRc, LRd, LRf and they all have the complete extracellular binding domain, but their intracellular tails differ; however, they all contain the two box domains. There is also a soluble form of leptin receptor in both humans and mice called LRe. In mice, LRe is directly secreted, while in humans, LRe is generated by ectodomain shedding (metalloproteases cut the receptor off the surface). **(B)** Leptin receptor isoforms are generated by alternative splicing or processing at the cell membrane. The long form of leptin receptor, also known as LRb, is the only known receptor variant that is capable of signaling through the JAK-STAT pathway. LRb has a long intracellular tail that includes several tyrosine residues that are phosphorylated for signal transduction by JAK2. LRb signaling primarily occurs through the JAK2/STAT3 pathway, with STAT3 translocating to the nucleus to modify gene expression. LRb also signals through the PI3K/Akt pathway and the MAPK pathway. These pathways in immune cells have been shown to lead to metabolic and functional changes, which could account for the pleiotropic effects of leptin on different immune cell types (56–58).

CD4^+^ T cells express high levels of the long isoform of the leptin receptor (Ob-Rb), which is significant because it is the only isoform that can signal through the Janus kinase (JAK)-signal transducer and activator of transcription (STAT) pathway (55), as shown in **Figure 1**. Leptin receptor signaling in T cells has been shown to promote survival, proliferation, cytokine production, and differentiation. *In vivo*, leptin treatment of wildtype (WT) mice was shown to inhibit steroid-induced apoptosis of lymphocytes (59). In response to leptin treatment, naïve CD4^+^ T cells, but not memory T cells, showed an increase in proliferation in a mixed lymphocyte reaction (35). In an older study of human cells, monocyte-depleted peripheral blood mononuclear cells (PBMCs) stimulated with phytohemagglutinin (PHA) and Concanavalin A (ConA) and treated with leptin had increased proliferation compared to untreated cells (60). More recent studies have demonstrated that CD4^+^ T cells from leptin receptor mutant (*db/db*) mice have reduced proliferation when compared to WT CD4^+^ T cells, suggesting that leptin signaling on CD4^+^ T cells is required for proliferation (31).

JAK-STAT signaling is downstream of many lymphocyte receptors that promote the production of various cytokines. Thus, as one would predict, leptin treatment of bulk, non-differentiated T cells influenced cytokine production by these cells. Leptin treatment of CD4^+^ T cells increased pro-inflammatory cytokine production, namely T helper 1 (Th1) cytokines interferon gamma (IFN-γ)and IL-2, while decreasing production of the T helper 2 (Th2) cytokine IL-4 (35). Moreover, activated CD4^+^ T cells generated from T cell specific leptin receptor conditional knockout mice were found to produce less IFN-γ than WT CD4^+^ T cells (31). Together, these data suggest that leptin promotes pro-inflammatory cytokine production in CD4^+^ T cells.

Leptin has also been shown to play a role in the differentiation of T cells into functional subsets. Hypoleptinemia induced by fasting has been shown to suppress the number of effector T cells, but not regulatory T cells (Treg cells) in mice. In fact, the same study found that while Treg proportions were increased in fasting, absolute numbers of Treg cells were unchanged, suggesting that leptin promotes the differentiation of effector T cells, but not Treg cells, and that any change in Treg cell proportions were indirect (34). In contrast, CD4^+^ T cells isolated from fasted hypoleptinemic mice had decreased differentiation into T helper 17 (Th17) cells *in vitro* compared to CD4^+^ T cells isolated from *ad lib* fed mice. When the fasted mice were given leptin injections twice daily, Th17 differentiation was restored, suggesting that leptin is critical for differentiation into Th17 cells (34). In support of this, Th17 differentiation *in vitro* was decreased in CD4^+^ T cells isolated from mice with T cell specific knockout of leptin receptor compared to WT controls (34). Furthermore, T cell specific leptin receptor knockout mice had decreased frequency of Th17 cells and increased frequency of Treg cells in the lamina propria (61).

The mechanism by which leptin promotes Th17 differentiation has been investigated. Leptin signaling promotes transcription of RAR-related orphan receptor gamma (RORγt), which is the critical transcription factor for Th17 fate. When RORγt-deficient CD4^+^ T cells were retrovirally transfected with a plasmid containing the *Rorc* gene, which encodes for RORγt, leptin treatment was shown to increase transcription of RORγt in these cells (62). This mechanism could also explain the inhibition of Treg differentiation by leptin, because Th17 and Treg cells have an antagonistic developmental program, where expression of the Th17 transcriptional program inhibits Treg development and vice versa, so that leptin promotion of Th17 fate by increasing RORγt transcription also directly inhibits Treg differentiation (63, 64). Given the pro-inflammatory effect of leptin on T cells, leptin is being investigated for use in cancer treatment to enhance the tumor-fighting action of T cells (65).

Interestingly, Treg cells express high amounts of leptin receptor, and have been shown to be capable of secreting leptin (66, 67). However, Treg cells are decreased in diet-induced obesity, which is consistent with the role of leptin in inhibiting Treg cell proportions, given that leptin levels are elevated in this setting (68). Treg cell proportions are also specifically decreased in the adipose tissue in diet-induced obesity, where leptin levels are expected to be highest (69). On the other hand, leptin mutant *ob/ob* mice were shown to have increased peripheral Foxp3+ CD4^+^ Treg cells compared to WT mice, further supporting the role of leptin, and not obesity alone, in decreasing Treg cell proportions (67). Leptin has also been shown to inhibit Treg cell proliferation in primary human cells, and blockade of leptin binding to Treg cells using anti-leptin antibodies led to increased Treg cell proliferation (67).

### B Cells

Leptin has been shown in both *ob/ob* mice and in fasting hypoleptinemic mice to be critical for normal B cell development in the bone marrow (70). Fasted mice and *ob/ob* mice both exhibited reduced proportions of pre-B, pro-B and immature B cells in bone marrow, which could be rescued by either intraperitoneal or intracerebroventricular injections of leptin (70). These findings demonstrate a possible central (neurological) mechanism as well as a peripheral mechanism by which leptin may promote B cell development (70).

Additionally, leptin has been shown to promote B cell homeostasis by inhibiting apoptosis and promoting cell cycle entry. B cells from *db/db* mice showed increased apoptosis compared to B cells from WT mice (37). Moreover, leptin treatment of WT B cells *in vitro* reduced apoptosis when B cells were treated with anti-IgM, CD40L, or LPS (37). Bcl-2 expression was upregulated upon leptin treatment, while anti-apoptotic members of the Bcl-2 family such as Bax, Bim and Bad were decreased, suggesting a possible mechanism for leptin’s effect on B cell survival (37). Leptin also promoted cell cycle entry by increasing the transcription of genes that regulate cell cycle, particularly in the presence of co-stimulation (37).

Human B cells stimulated with leptin *in vitro* were shown to exhibit a more pro-inflammatory phenotype characterized by increased expression of inflammatory cytokines IL-6 and TNF, as well as toll-like receptor 4 (TLR4), a pattern recognition receptor that recognizes lipopolysaccharide (LPS) found on gram-negative bacteria (71). These B cells also showed reduced class switching and IgG production in response to leptin, suggesting that while they may be more inflammatory, they do not necessarily have increased function (71). These findings are supported by another study that showed human peripheral blood B cells have increased IL-6, TNF, and IL-10 production when treated with leptin *in vitro* (72). This study further demonstrated that leptin signaling in B cells activated JAK2, STAT3, ERK1/2, and p38 MAPK pathways (72). Inhibiting these signaling molecules decreased IL-6, TNF, and IL-10 production following leptin treatment, demonstrating that signaling through JAK2, STAT3, ERK1/2 and p38 MAPK is required to increase cytokine production in response to leptin (72). Similar findings were described in B cells from obese patients, suggesting that the phenotype of inflammatory B cells in obesity may be mediated, at least in part, by leptin signaling (38, 73).

## Innate Immune Cells

Leptin has been shown to have a generally pro-inflammatory effect on innate immune cells, but with distinct effects on each innate immune cell type, as discussed below.

### Macrophages and Monocytes

Macrophages are key regulators of adipose tissue inflammation in obesity and, therefore, the effects of leptin on macrophages is highly relevant in the setting of diet-induced obesity. Bone marrow derived macrophages from leptin receptor mutant *db/db* mice showed decreased phagocytosis and decreased inflammatory cytokine production in response to LPS treatment *in vitro* (39). In leptin mutant *ob/ob* mice, bone marrow derived macrophages were shown to have decreased phagocytic ability *in vitro*, and *ob/ob* mice failed to clear infections such as *Escherichia Coli* and *Klebsiella pneumonia in vivo* (39, 74). Obese Zucker (*fa/fa*) rats with a leptin receptor mutation, had reduced ability to clear the fungal infection *Candida albicans in vivo*, as measured by colony-forming units in lung, liver, spleen, heart, and kidney (75). Furthermore, mice with macrophage-specific deletion of the leptin receptor had impaired clearance of *Streptococcus pneumoniae* in the lungs and spleen (40). The same macrophage specific leptin receptor knockout mice also had elevated pulmonary IL-13 and TNF compared to WT mice 48 h after infection with *S. pneumoniae* (40). Complementary *in vitro* studies of alveolar macrophages from macrophage specific leptin receptor knockout mice likewise showed decreased macrophage killing and phagocytosis (40). Thus, leptin acts specifically on macrophages *via* the leptin receptor to promote both phagocytosis and cytokine production (40).

Monocytes are innate immune cells that can differentiate into tissue-specific macrophages and myeloid-derived dendritic cells. Primary human monocytes from PBMCs and THP-1 monocytes, a human monocyte cell line, have been shown to increase toll-like receptor 2 (TLR2) expression in response to leptin treatment *in vitro* (41). TLR2 is a pattern recognition receptor that allows innate immune cells to recognize pathogens. By promoting TLR2 expression on monocytes, leptin is able to promote the innate immune response to pathogens such as *E. coli*. In human studies, leptin treatment of monocytes isolated from PBMCs increased the production of type 1 cytokines, including IL-1β, IL-6, and TNF, and resistin (42). Like in T cells, leptin appears to promote an inflammatory phenotype in monocytes.

### Mast Cells

Another innate immune cell that has been shown to respond to leptin is the mast cell. Mast cells are best known for their roles in allergic response and protecting against helminth infection. Leptin mutant *ob/ob* mice showed decreased percentage of mast cells in inguinal adipose tissue, but did not show mast cell deficiencies in other tissues (76). Several studies have proposed a role for mast cells in polarization of macrophages by secretion of cytokines (77). For example, IL-33 treatment of mast cells causes production of IL-6 and IL-13, which are cytokines known to promote alternatively activated macrophages that suppresses T cell inflammation (77). One group has investigated the role of leptin in mast cell function and the subsequent effect on macrophages in the context of obesity (43). In this study, mast cells derived from WT bone marrow (BMMCs) were co-cultured with bone marrow-derived macrophages (BMDMs) from leptin receptor mutant *db/db* mice, in the presence or absence of leptin. Leptin treatment of the mast cells led to increased macrophage production of IFN-γ (43). In the same study, leptin inhibited the anti-inflammatory M2-like macrophage phenotype by decreasing arginase-1 and IL-10 expression (43). Mast cells from leptin mutant *ob/ob* mice, on the other hand, promoted maturation of WT macrophages to an M2-like anti-inflammatory phenotype when they were co-cultured *in vitro*, suggesting that leptin production by mast cells may be important in promoting a pro-inflammatory macrophage phenotype (43). Mast cells are also known to play a role in adipose tissue remodeling in obesity, promoting the inflammatory phenotype of adipose tissue by secreting inflammatory molecules such as TNF and pro-angiogenesis molecules such as chymase (78).

### Dendritic Cells

Dendritic cells (DCs) function at the interface of the innate and adaptive immune system by uptaking, processing, and presenting antigens to T cells. DCs were shown to express leptin receptor, both at the protein and mRNA level, which signals through STAT3 upon stimulation (44). Furthermore, leptin was found to have an anti-apoptotic effect on DCs *in vitro* by increasing expression of the anti-apoptotic proteins Bcl-2 and Bcl-xL (44). Mature DCs are more capable of stimulating an appropriate and strong T cell response; at homeostasis, leptin promoted DC maturation and function (45). Leptin treatment of DCs increased production of IL-1β, IL-6, IL-12, TNF, and MIP-1α (44). DCs generated from the bone marrow of leptin mutant *ob/ob* mice (BMDCs) showed reduced expression of MHC-II, CD80, CD86, and CD40 (45). MHC-II and CD80/86, in particular, are critical for activating CD4^+^ T cells, and CD4^+^ T cells stimulated in co-culture by BMDCs from *ob/ob* or *db/db* mice produced less IFN-γ and proliferated less than CD4^+^ T cells stimulated by BMDCs from WT mice (45). Furthermore, BMDCs from *ob/ob* mice produced less IL-6, IL-12, and TNF after two days of maturation (45).

### Neutrophils, Basophils, and Eosinophils

Neutrophils are some of the best studied innate immune cells with regard to leptin response. Interestingly, neutrophils only express the short form leptin receptor, which lacks JAK-STAT signaling (79), as shown in **Figure 1**. Leptin has been shown to inhibit neutrophil apoptosis, suggesting that leptin acts as a survival factor for neutrophils (46). Leptin also acts like a chemoattractant for neutrophils in the wildtype setting (47). *In vitro*, WT neutrophils from bone marrow (isolated by density gradient) were shown to exhibit chemotaxis toward leptin, whereas neutrophils from mice with a leptin receptor variant (Q223R) show reduced chemotaxis toward leptin (22, 47). In various infection models, leptin receptor deficiency (*db/db* mice) was shown to reduce neutrophil trafficking to the site of infection (80, 81). In a model of LPS-induced lung injury, neutrophil trafficking to the lungs was impaired in *db/db* mice, as demonstrated by reduced numbers of neutrophils in the airways (BAL), while there was increased neutrophilia in the blood (81). In a model of *Clostridium difficile* colitis, leptin receptor STAT3 mutant mice (S1138) showed decreased neutrophil numbers in the lamina propria following infection (80). Furthermore, leptin administration by oropharyngeal aspiration was shown to promote neutrophil trafficking to the lungs after *E. coli* infection as determined by neutrophil numbers in bronchoalveolar lavage fluid (47). Overall, it appears that leptin primarily acts as a chemoattractant for neutrophils, particularly during infection in the lung. Polymorphonuclear neutrophils (PMNs) isolated from human blood were shown to increase their production of oxidative species after leptin treatment *in vitro*, which the authors propose would promote bacterial clearance (48). This data points to leptin promoting neutrophil function as well as chemotaxis.

Basophils and eosinophils have also been shown to express leptin receptor (49, 50). Leptin has been shown to be a survival factor for both eosinophils and basophils (49, 50). Similar to neutrophils, leptin has also been shown to act as a chemoattractant for both basophils and eosinophils. Basophils and eosinophils isolated from human blood migrated in a dose dependent manner toward leptin *in vitro* in a transwell system or similar experimental setup (49, 51, 82). Additionally, leptin promoted basophil and eosinophil trafficking toward other chemoattractants, such as eotaxin (49, 82). Specifically, human basophils exposed to leptin demonstrated increased migration *in vitro* toward eotaxin (49). Human eosinophils were pre-treated *in vitro* with leptin for 1 h prior to assessing the migration of eosinophils toward eotaxin; more leptin treated eosinophils migrated toward eotaxin than untreated eosinophils (51). Given that leptin promotes type 1 cytokine production in other immune cells, leptin treatment of basophils had a slightly counter-intuitive result in that basophils increased type 2 cytokine production, including IL-4 and IL-13 (49).

### NK Cells and ILCs

At the interface between adaptive and innate immunity sit natural killer (NK) cells and innate lymphoid cells (ILCs). These cells are able to respond to pathogens with rapid cytokine production and, in the case of NK cells, killing of infected cells. NK cells and ILCs are part of a complex family of lymphocytes that have phenotypic characteristics that mirror CD4^+^ and CD8^+^ T cell families, and are currently under intense study. In the leptin receptor mutant *db/db* mouse, NK percentage and number were found to be decreased in spleen, liver, lung, and blood (83). This indicates that leptin receptor is required for normal NK cell development. When NK cells from *db/db* mice were activated by poly I:C, fewer NK cells expressed CD69, an early NK cell activation marker. This indicates that leptin receptor is required for rapid activation of NK cells (83). The nuances of NK cell response to leptin treatment appear to be extremely dependent on dose and length of exposure. Brief treatment (20 min) of human NK cells with leptin increased NK cell cytotoxicity as measured by a chromium release assay (52), and 18-h leptin treatment increased human NK cell IFN-γ and perforin production, as well as inflammatory markers, such as TRAIL (52, 53). Long exposure (72 h) to leptin, however, inhibited NK cell production of IFN-γ, as measured by ELISA, and cytotoxicity, as measured by chromium release assay (52).

Leptin was shown to promote ILC2 and Th2 cytokine production in allergic airway disease, demonstrating that increased leptin levels associated with obesity could be driving the increased risk for allergy/asthma that is observed in obesity (54). While a Th2-type phenotype is not considered pro-inflammatory, this is another example of how leptin can license immune cells to perform their functions, even in tissues outside of adipose.

## Mechanisms of Leptin Effects on Immune Cells

The downstream effects of leptin receptor signaling have been best studied in CD4^+^ T cells, where leptin signaling promotes a measurable and direct effect on cellular metabolism.

### Leptin Receptor Signaling

The mechanism of leptin’s actions on immune cells is complex, in part because leptin receptor has several isoforms generated though alternative splicing, which each have differing signaling capacities (84), as shown in **Figure 1**. For example, T cells express the long form of the leptin receptor, particularly after activation, while neutrophils only express the short form, and NK cells express both the short and long form receptors (85). These isoforms differ primarily in the intracellular domain responsible for downstream signaling. While both the short and long receptor isoforms are capable of transmitting some signals inside the cell, it is believed that only the long form has complete signaling capabilities.

The long form of the receptor contains fully functional JAK2 binding sites, and upon leptin binding, the leptin receptor has been shown to homodimerize, bind to, and phosphorylate JAK2 (84). STAT proteins are then recruited to the receptor complex and phosphorylated, which leads to STAT dimerization, translocation to the nucleus, and binding to promoter sites. The system is highly regulated, as this signaling also leads to transcription of SOCS3, which is a negative regulator of the JAK/STAT signaling cascade. Leptin receptor can also signal through the PI3K/Akt and MAPK pathways through IRS-1/2 and SHP-2 recruitment, respectively (86).

### Leptin Effects on Cellular Metabolism

It is now clear that leptin signaling through leptin receptor promotes a metabolic change in CD4^+^ T cells. Since immune cell metabolism and function are intimately related, recent work has investigated if leptin-induced changes in CD4^+^ T cell function are mediated by changes in T cell metabolism (87). This was first explored in a fasting model of hypoleptinemia. CD4^+^ T cells isolated from fasted mice and activated *in vitro* showed decreased glucose uptake and decreased glycolytic rate compared to CD4^+^ T cells isolated from *ad lib* fed control mice, suggesting that leptin signaling promotes glycolytic metabolism in CD4^+^ T cells (31). As glycolytic metabolism is strongly associated with inflammatory function, this fits with the previously discussed role of leptin in promoting inflammatory cytokine production in CD4^+^ T cells (31, 34). CD4^+^ T cells isolated from leptin receptor mutant *db/db* mice also showed reduced glucose uptake, in part secondary to decreased glucose transporter Glut1 expression, and decreased glycolytic rate compared to WT CD4^+^ T cells when activated *in vitro*. Additionally, CD4^+^ T cells from *db/db* mice were less metabolically active with decreased extracellular acidification rate (ECAR), a measure of lactate production downstream of glycolysis, as well as decreased oxygen consumption rate, a measure of mitochondrial oxidation (31). These studies indicate that leptin receptor signaling in T cells leads to changes in cellular metabolism.

The functional subsets of CD4^+^ T cells have distinct metabolic characteristics, and leptin influences the metabolism of these subsets in different ways. CD4^+^ T cells were isolated from WT mice that were either fed *ad lib*, fasted for 48 h to promote hypoleptinemia, or fasted while receiving twice daily intraperitoneal leptin injections, and differentiated *in vitro* into Th17 or Treg cells. Th17 cells generated from fasted mice showed decreased ECAR and oxygen consumption rate (OCR), but this was rescued when fasted mice received leptin injections (34). In contrast, Treg cell metabolism was not impacted by fasting (34). To investigate the direct role of leptin signaling on T cell metabolism, CD4^+^ T cells were isolated from T cell specific leptin receptor conditional knockout mice or WT controls and differentiated into Th17 or Treg cells *in vitro* (34). Th17 cells from leptin receptor knockout mice, but not Treg cells, showed decreased expression of key metabolic genes Glut1 and hexokinase 2 (HK2), which is a rate-limiting enzyme of glycolysis (34). Th17 cells from leptin receptor knockout mice also had decreased glucose uptake and lactate production compared to Th17 cells from WT controls, suggesting that leptin signaling promotes appropriate Th17 cells glycolytic signaling to fuel Th17 cell function (34). Combined, these data suggest that leptin has a T cell intrinsic effect on metabolism that promotes glycolytic and oxidative metabolism necessary for proper T cell function.

## Role of Leptin in Immune-Mediated Disease

Leptin has been implicated in a number of immune-mediated diseases, many of which are also associated with obesity. These range from type 2 diabetes to autoimmune disease to infection. In this section, we will explore the role that leptin plays in mediating the immune response in obesity-associated disease.

### Metabolic Disease: Type 2 Diabetes

The incidence of type 2 diabetes mellitus (T2DM) is increasing in parallel with the prevalence of obesity. Obesity-associated inflammation has been shown to drive insulin resistance, leading to T2DM (56). Methods that eliminate the inflammatory T cell or macrophage response in obesity prevent insulin resistance and progression to T2DM. For example, several immunocompromised mouse models (NOD and SCID mice) have been found to be resistant to the development of obesity and insulin resistance when fed high fat diet (88). Elimination of CD11c+ macrophages in a mouse model of obesity resulted in increased insulin sensitivity (89), and a less specific macrophage deletion strategy using chlodronate liposomes leading to apoptosis of phagocytic cells also resulted in increased insulin sensitivity and improved systemic glucose tolerance (90). T cell-deficient TCR-knockout mice that lack CD4^+^ and CD8^+^ T cells had decreased obesity-induced macrophage infiltration and decreased insulin resistance on high fat diet compared to wildtype controls (91), and obese mice that lack IFN-γ had improved insulin sensitivity compared to obese wildtype controls (92). Similarly, knockout of the Th1-associated transcription factor T-bet improved insulin sensitivity in high-fat diet fed mice (93). Based on the pro-inflammatory effect of leptin on immune cells as described above, it is possible that obesity-associated hyperleptinemia is responsible, at least in part, for promoting the obesity-associated inflammation that leads to insulin resistance and diabetes in obesity.

### Autoimmunity

In addition to metabolic syndrome and T2DM, obesity predisposes patients to select autoimmune and inflammatory diseases such as multiple sclerosis (MS), rheumatoid arthritis, and systemic lupus erythematosus (1, 2). Leptin deficiency has been shown in mice to protect against experimental autoimmune encephalomyelitis (EAE) (94), colitis (95), T cell mediated hepatitis (96), and glomerulonephritis (97). One key example is the well-studied autoimmune model EAE, a mouse model of MS. Leptin has been shown to play a critical role in EAE progression, and leptin mutant *ob/ob* mice are protected from development of EAE (94). Furthermore, EAE disease scores were reduced when anti-leptin antibodies were administered either before or after the induction of EAE in mice (98).

Since inflammatory Th17 cells play an important role in the pathogenesis of EAE, and leptin is known to promote Th17 cell differentiation, the role of leptin signaling on T cells in EAE was investigated. T cell specific leptin receptor knockout mice were protected from EAE compared to WT mice, with lower disease scores (61). Furthermore, the cytokine profile of mice treated with anti-leptin antibodies was changed to a non-inflammatory Th2/Treg cytokine profile (IL-4, IL-10) instead of the pro-inflammatory Th1/Th17 cytokine profile typically seen in EAE (98). Blocking leptin also decreased proliferation of antigen specific T cells in this autoimmune model (98). These studies indicate a specific role for leptin in promoting inflammatory T cell proliferation and function that promotes EAE disease progression.

In a model of fasting-induced hypoleptinemia, C57BL/6 mice fasted for 48 h had lower disease scores than *ad lib* fed mice following EAE induction, but this effect was reversed by exogenous leptin treatment administered during the fasting period (34). This demonstrates that leptin alone is sufficient to license the development of autoimmunity in undernourished mice that were otherwise protected against disease. In the same study, Th17 cells from fasted mice undergoing EAE induction had decreased expression of the key glycolytic protein HK2 as well as decreased expression of the glycolysis-promoting regulator HIF-1α, and both HK2 and HIF-1α levels were normalized when fasted mice were treated with leptin. In human studies, serum leptin levels were found to be increased prior to onset of clinical symptoms in relapsing-remitting MS, indicating that leptin may both contribute to the pathogenesis of MS and be a useful marker of disease (99, 100).

### Infection

The link between leptin and susceptibility to infection has been studied in animal models. Leptin mutant *ob/ob* mice were shown to be more susceptible to death by LPS stimulation, and leptin treatment was shown to partially reverse this effect (101, 102). Interestingly, LPS and other inflammatory signals have been shown to induce leptin production from adipose tissue (103–106). It is possible that this increase in leptin can then stimulate the inflammatory response necessary to fight the infection that LPS is modeling.

Many studies have examined the effect of leptin treatment on various bacterial models of infection in mice. Leptin universally decreased bacterial load and improved survival or immune response to infection with *Mycobacterium tuberculosis*, *Klebsiella pneumonia*, and *Pneumococcal pneumonia* (107). These data indicate that leptin is important for promoting the proper immune response to clear bacterial infections.

Leptin receptor mutant *db/db* mice also had reduced survival and impaired viral clearance when infected with influenza virus, as well as reduced IFN-γ production in the lungs following infection (108). Interestingly, when lung epithelium or alveolar macrophages, specifically, were deficient in leptin receptor, the mice cleared virus better than global leptin receptor knock out mice (108). These data indicate that in influenza infection, the response to leptin of other immune cells, such as T cells, B cells or NK cells, is key to clearing virus.

## Leptin Studies in Humans

Congenital leptin deficiency in humans, while rare, can provide important information regarding the role of leptin. Genetic mutations in both the leptin gene and the gene for leptin receptor have been described, and these genetic variants cause similar phenotypes in terms of immune response. Mutations in leptin or the leptin receptor gene cause early onset extreme obesity, hyperphagia, hypogonadism, and metabolic disorders (109). Furthermore, these patients develop repeat infections, and humans with leptin deficiency are at increased risk of death due to intracellular infections (28). Leptin replacement therapy has been shown in humans to increase CD4^+^ T cell numbers and reverse defects in CD4^+^ T cell proliferation and cytokine production (110). These data clearly underscore the importance of leptin in normal immune function and protection from infection. Consistent with this, fasting reduces leptin levels and leads to reduced lymphocyte counts in the blood (111).

On the other hand, obesity is also associated with increased morbidity and mortality in response to select infections such as bacterial cellulitis (112), influenza (113–117), and coronavirus (118–124), although the role for leptin in this setting has not been determined. While the etiology of obesity is complex, it is possible that increased leptin signaling promotes excessive inflammation and potentially cytokine storm.

## Conclusion

Leptin is a pleiotropic adipokine with diverse effects on cell types throughout the body. Its role in neuroendocrine signaling, homeostasis, and metabolism has been well studied. More recently, leptin has been identified as an important immune modulator with a wide range of functions, many of which are pro-inflammatory. The complexity of leptin receptor signaling, as well as the several variants of the receptor with unique signaling capabilities likely allows for the diversity of effects that are mediated on distinct immune cells, sometimes located within the same tissues. Overall, it is clear that leptin plays a critical role in obesity-associated inflammation by promoting pro-inflammatory immune phenotypes. While leptin has not been successful in treating obesity as a weight loss drug, it is possible that targeting leptin or leptin signaling could be therapeutic for autoimmune disease or the low-grade, chronic inflammation associated with obesity and metabolic syndrome.

## Author Contributions

Both KK and NM contributed to the writing and editing of the manuscript. All authors contributed to the article and approved the submitted version.

## Funding

This work was supported by the National Institutes of Health (R01-DK106090).

## Conflict of Interest

The authors declare that the research was conducted in the absence of any commercial or financial relationships that could be construed as a potential conflict of interest.

## References

[1] Kopelman P Health risks associated with overweight and obesity. Obesity Rev (2007) 8(s1):13–7. 10.1111/j.1467-789X.2007.00311.x 17316295

[2] Haslam DW James WP Obesity. Lancet (2005) 366(9492):1197–209. 10.1016/S0140-6736(05)67483-1 16198769

[3] Alwarawrah Y Kiernan K MacIver NJ Changes in Nutritional Status Impact Immune Cell Metabolism andFunction. Front Immunol (2018)9:1055–69. 10.3389/fimmu.2018.01055 PMC5968375 29868016

[4] Kern PA Saghizadeh M Ong JM Bosch RJ Deem R Simsolo RB The expression of tumor necrosis factor in human adipose tissue. Regulation by obesity, weight loss, and relationship to lipoprotein lipase. J Clin Invest (1995) 95(5):2111–9. 10.1172/JCI117899 PMC295809 7738178

[5] Hotamisligil GS Arner P Caro JF Atkinson RL Spiegelman BM Increased adipose tissue expression of tumor necrosis factor-alpha in human obesity and insulin resistance. J Clin Invest (1995) 95(5):2409–15. 10.1172/JCI117936 PMC295872 7738205

[6] Hotamisligil GS Shargill NS Spiegelman BM Adipose expression of tumor necrosis factor-alpha: direct role in obesity-linked insulin resistance. Science (New York NY) (1993) 259(5091):87. 10.1126/science.7678183 7678183

[7] Pickup JC Mattock MB Chusney GD Burt D NIDDM as a disease of the innate immune system: association of acute-phase reactants and interleukin-6 with metabolic syndrome X. Diabetologia (1997) 40(11):1286. 10.1007/s001250050822 9389420

[8] Kern PA Ranganathan S Li C Wood L Ranganathan G Adipose tissue tumor necrosis factor and interleukin-6 expression in human obesity and insulin resistance. Am J Physiol-Endocrinol Metab (2001) 280(5):E745–E51. 10.1152/ajpendo.2001.280.5.E745 11287357

[9] Xu E Pereira MMA Karakasilioti I Theurich S Al-Maarri M Rappl G Temporal and tissue-specific requirements for T-lymphocyte IL-6 signalling in obesity-associated inflammation and insulin resistance. Nat Commun (2017) 8(1):14803. 10.1038/ncomms14803 28466852 PMC5418621

[10] van Meijel RLJ Blaak EE Goossens GH Chapter 1 - Adipose tissue metabolism and inflammation in obesity. In: Johnston RA Suratt BT, editors. Mechanisms and Manifestations of Obesity in Lung Disease. Academic Press (2019). p. 1–22.

[11] Berg Anders H Scherer Philipp E Adipose Tissue, Inflammation, and Cardiovascular Disease. Circ Res (2005) 96(9):939–49. 10.1161/01.RES.0000163635.62927.34 15890981

[12] Kanda H Tateya S Tamori Y Kotani K Hiasa K-I Kitazawa R MCP-1 contributes to macrophage infiltration into adipose tissue, insulin resistance, and hepatic steatosis in obesity. J Clin Invest (2006) 116(6):1494–505. 10.1172/JCI26498 PMC1459069 16691291

[13] Nishimura S Manabe I Nagasaki M Eto K Yamashita H Ohsugi M CD8+ effector T cells contribute to macrophage recruitment and adipose tissue inflammation in obesity. Nat Med (2009) 15(8):914–20. 10.1038/nm.1964 19633658

[14] Jin Young H Yoon Jeong P Mira H Jae Bum K Crosstalk between Adipocytes and Immune Cells in Adipose Tissue Inflammation and Metabolic Dysregulation in Obesity. Mol Cells (2014) 37(5):365–71. 10.14348/molcells.2014.0074 PMC4044307 24781408

[15] Cipolletta D Feuerer M Li A Kamei N Lee J Shoelson SE PPAR-γ is a major driver of the accumulation and phenotype of adipose tissue Treg cells. Nature (2012) 486(7404):549–53. 10.1038/nature11132 PMC3387339 22722857

[16] Lynch L O’Shea D Winter DC Geoghegan J Doherty DG O’Farrelly C Invariant NKT cells and CD1d+ cells amass in human omentum and are depleted in patients with cancer and obesity. Eur J Immunol (2009) 39(7):1893–901. 10.1002/eji.200939349 19585513

[17] Gerriets VA MacIver NJ Role of T Cells in Malnutrition and Obesity. Front Immunol (2014) 5:379–90. 10.3389/fimmu.2014.00379 PMC4127479 25157251

[18] Winer S Chan Y Paltser G Truong D Tsui H Bahrami J Normalization of obesity-associated insulin resistance through immunotherapy. Nat Med (2009) 15(8):921–9. 10.1038/nm.2001 PMC3063199 19633657

[19] Feuerer M Herrero L Cipolletta D Naaz A Wong J Nayer A Lean, but not obese, fat is enriched for a unique population of regulatory T cells that affect metabolic parameters. Nat Med (2009) 15(8):930–9. 10.1038/nm.2002 PMC3115752 19633656

[20] Weisberg SP McCann D Desai M Rosenbaum M Leibel RL Ferrante AW Jr. Obesity is associated with macrophage accumulation in adipose tissue. J Clin Invest (2003) 112(12):1796–808. 10.1172/JCI19246 PMC296995 14679176

[21] Makki K Froguel P Wolowczuk I Adipose tissue in obesity-related inflammation and insulin resistance: cells, cytokines, and chemokines. ISRN Inflamm (2013) 2013:139239–. 10.1155/2013/139239 PMC3881510 24455420

[22] Naylor C Burgess S Madan R Buonomo E Razzaq K Ralston K Leptin receptor mutation results in defective neutrophil recruitmentto the colon during Entamoeba histolytica infection. mBio(2014) 5(6):1–8. 10.1128/mBio.02046-14 PMC4271549 25516614

[23] Soliman AT ElZalabany MM Salama M Ansari BM Serum leptin concentrations during severe protein-energymalnutrition: correlation with growth parameters and endocrine function. Metabolism (2000) 49(7):819–25. 10.1053/meta.2000.6745. 10909989

[24] Korbonits M Trainer PJ Little JA Edwards R Kopelman PG Besser GM Leptin levels do not change acutely with food administration innormal or obese subjects, but are negatively correlated with pituitary-adrenalactivity. Clin Endocrinol (Oxf). (1997) 46(6):751-7. 10.1046/j.1365-2265.1997.1820979.x 9274707

[25] Park H-K Ahima RS Physiology of leptin: energy homeostasis, neuroendocrine function and metabolism. Metabolism (2015) 64(1):24–34. 10.1016/j.metabol.2014.08.004 25199978 PMC4267898

[26] Khan SM Hamnvik O-PR Brinkoetter M Mantzoros CS Leptin as a modulator of neuroendocrine function in humans. Yonsei Med J (2012) 53(4):671–9. 10.3349/ymj.2012.53.4.671 PMC3381496 22665330

[27] Rosenbaum M Leibel RL 20 years of leptin: role of leptin in energy homeostasis in humans. J Endocrinol (2014) 223(1):T83–96. 10.1530/JOE-14-0358 PMC4454393 25063755

[28] Ozata M Ozdemir IC Licinio J Human leptin deficiency caused by a missense mutation: multiple endocrine defects, decreased sympathetic tone, and immune system dysfunction indicate new targets for leptin action, greater central than peripheral resistance to the effects of leptin, and spontaneous correction of leptin-mediated defects. J Clin Endocrinol Metab (1999) 84(10):3686–95. 10.1210/jcem.84.10.5999 10523015

[29] Gainsford T Willson TA Metcalf D Handman E McFarlane C Ng A Leptin can induce proliferation, differentiation, and functional activation of hemopoietic cells. Proc Natl Acad Sci U S A (1996) 93(25):14564–8. 10.1073/pnas.93.25.14564 PMC26173 8962092

[30] Mandel MA Mahmoud AA Impairment of cell-mediated immunity in mutation diabetic mice (db/db). J Immunol (1978) 120(4):1375–7. 347001

[31] Saucillo DC Gerriets VA Sheng J Rathmell JC Maciver NJ Leptin metabolically licenses T cells for activation to link nutrition and immunity. J Immunol (2014) 192(1):136–44. 10.4049/jimmunol.1301158 PMC3872216 24273001

[32] Kim SY Lim JH Choi SW Kim M Kim ST Kim MS Preferential effects of leptin on CD4 T cells in central and peripheral immune system are critically linked to the expression of leptin receptor. Biochem Biophys Res Commun (2010) 394(3):562–8. 10.1016/j.bbrc.2010.03.019 20227394

[33] Procaccini C Jirillo E Matarese G Leptin as an immunomodulator. Mol Aspects Med (2012) 33(1):35–45. 10.1016/j.mam.2011.10.012 22040697

[34] Gerriets VA Danzaki K Kishton RJ Eisner W Nichols AG Saucillo DC Leptin directly promotes T-cell glycolytic metabolism to drive effector T-cell differentiation in a mouse model of autoimmunity. Eur J Immunol (2016) 46(8):1970–83. 10.1002/eji.201545861 PMC5154618 27222115

[35] Lord GM Matarese G Howard JK Baker RJ Bloom SR Lechler RI Leptin modulates the T-cell immune response and reverses starvation-induced immunosuppression. Nature (1998) 394(6696):897–901. 10.1038/29795 9732873

[36] Martín-Romero C Santos-Alvarez J Goberna R Sánchez-Margalet V Human Leptin Enhances Activation and Proliferation of Human Circulating T Lymphocytes. Cell Immunol (2000) 199(1):15–24. 10.1006/cimm.1999.1594 10675271

[37] Lam QLK Wang S Ko OKH Kincade PW Lu L Leptin signaling maintains B-cell homeostasis via induction of Bcl-2 and Cyclin D1. Proc Natl Acad Sci (2010) 107(31):13812. 10.1073/pnas.1004185107 20643953 PMC2922219

[38] Frasca D Blomberg BB Adipose Tissue Inflammation Induces B Cell Inflammation and Decreases B Cell Function in Aging. Front Immunol (2017) 8:1003. 10.3389/fimmu.2017.01003 28894445 PMC5581329

[39] Loffreda S Yang SQ Lin HZ Karp CL Brengman ML Wang DJ Leptin regulates proinflammatory immune responses. FASEB J (1998) 12(1):57–65. 10.1096/fasebj.12.1.57 9438411

[40] Mancuso P Curtis JL Freeman CM Peters-Golden M Weinberg JB Myers MG Jr. Ablation of the leptin receptor in myeloid cells impairs pulmonary clearance of Streptococcus pneumoniae and alveolar macrophage bactericidal function. Am J Physiol Lung Cell Mol Physiol (2018) 315(1):L78–l86. 10.1152/ajplung.00447.2017 29565180 PMC6087898

[41] Jaedicke KM Roythorne A Padget K Todryk S Preshaw PM Taylor JJ Leptin up-regulates TLR2 in human monocytes. J Leukoc Biol (2013) 93(4):561–71. 10.1189/jlb.1211606 23341537

[42] Tsiotra PC Boutati E Dimitriadis G Raptis SA High insulin and leptin increase resistin and inflammatory cytokine production from human mononuclear cells. BioMed Res Int (2013) 2013:487081. 10.1155/2013/487081 23484124 PMC3591160

[43] Zhou Y Yu X Chen H Sjöberg S Roux J Zhang L Leptin Deficiency Shifts Mast Cells toward Anti-Inflammatory Actions and Protects Mice from Obesity and Diabetes by Polarizing M2 Macrophages. Cell Metab (2015) 22(6):1045–58. 10.1016/j.cmet.2015.09.013 PMC4670585 26481668

[44] Mattioli B Straface E Quaranta MG Giordani L Viora M Leptin Promotes Differentiation and Survival of Human Dendritic Cells and Licenses Them for Th1 Priming. J Immunol (2005) 174(11):6820. 10.4049/jimmunol.174.11.6820 15905523

[45] Moraes-Vieira PM Larocca RA Bassi EJ Peron JP Andrade-Oliveira V Wasinski F Leptin deficiency impairs maturation of dendritic cells and enhances induction of regulatory T and Th17 cells. Eur J Immunol (2014) 44(3):794–806. 10.1002/eji.201343592 24271843 PMC4973395

[46] Bruno A Conus S Schmid I Simon H-U Apoptotic Pathways Are Inhibited by Leptin Receptor Activation in Neutrophils. J Immunol (2005) 174(12):8090. 10.4049/jimmunol.174.12.8090 15944317

[47] Ubags ND Vernooy JH Burg E Hayes C Bement J Dilli E The role of leptin in the development of pulmonary neutrophilia in infection and acute lung injury. Crit Care Med (2014) 42(2):e143–51. 10.1097/ccm.0000000000000048 PMC3947045 24231757

[48] Caldefie-Chezet F Poulin A Tridon A Sion B Vasson MP Leptin: a potential regulator of polymorphonuclear neutrophil bactericidal action? J Leukoc Biol (2001) 69(3):414–8. 11261788

[49] Suzukawa M Nagase H Ogahara I Han K Tashimo H Shibui A Leptin enhances survival and induces migration, degranulation, and cytokine synthesis of human basophils. J Immunol (2011) 186(9):5254–60. 10.4049/jimmunol.1004054 21421855

[50] Conus S Bruno A Simon HU Leptin is an eosinophil survival factor. J Allergy Clin Immunol (2005) 116(6):1228–34. 10.1016/j.jaci.2005.09.003 16337450

[51] Kato H Ueki S Kamada R Kihara J Yamauchi Y Suzuki T Leptin Has a Priming Effect on Eotaxin-Induced Human Eosinophil Chemotaxis. Int Arch Allergy Immunol (2011) 155(4):335–44. 10.1159/000321195 21346363

[52] Wrann CD Laue T Hübner L Kuhlmann S Jacobs R Goudeva L Short-term and long-term leptin exposure differentially affect humannatural killer cell immune functions. American Journal ofPhysiology-Endocrinology and Metabolism (2012)302(1): E108–16. 10.1152/ajpendo.00057.2011 21952038

[53] Lamas B Goncalves-Mendes N Nachat-Kappes R Rossary A Caldefie-Chezet F Vasson M-P Leptin modulates dose-dependently the metabolic and cytolyticactivities of NK-92 cells. J Cell Physiol (2013) 228(6):1202-9. 10.1002/jcp.24273. 23129404

[54] Zheng H Zhang X Castillo EF Luo Y Liu M Yang XO Leptin Enhances TH2 and ILC2 Responses in Allergic Airway Disease. J Biol Chem (2016) 291(42):22043–52. 10.1074/jbc.M116.743187 PMC5063987 27566543

[55] Cioffi JA Shafer AW Zupancic TJ Smith-Gbur J Mikhail A Platika D Novel B219/OB receptor isoforms: possible role of leptin in hematopoiesis and reproduction. Nat Med (1996) 2(5):585–9. 10.1038/nm0596-585 8616721

[56] Francisco V Pino J Campos-Cabaleiro V Ruiz-Fernández C Mera A Gonzalez-Gay MA Obesity, Fat Mass and Immune System: Role for Leptin. Front Physiol (2018) 9:640. 10.3389/fphys.2018.00640 29910742 PMC5992476

[57] Wauman J Zabeau L Tavernier J The Leptin Receptor Complex: Heavier Than Expected?Front Endocrinol (2017) 8:30–50. 10.3389/fendo.2017.00030 PMC5318964 28270795

[58] Frank P Lennart Z Kedar M Savvas NS Jan T 20 YEARS OF LEPTIN: Insights into signaling assemblies of the leptin receptor. J Endocrinol (2014) 223(1):T9–T23. 10.1530/JOE-14-0264 25063754

[59] Fujita Y Murakami M Ogawa Y Masuzaki H Tanaka M Ozaki S Leptin inhibits stress-induced apoptosis of T lymphocytes. Clin Exp Immunol (2002) 128(1):21–6. 10.1046/j.1365-2249.2002.01797.x PMC1906378 11982586

[60] Martin-Romero C Santos-Alvarez J Goberna R Sanchez-Margalet V Human leptin enhances activation and proliferation of human circulating T lymphocytes. Cell Immunol (2000) 199(1):15–24. 10.1006/cimm.1999.1594 10675271

[61] Reis BS Lee K Fanok MH Mascaraque C Amoury M Cohn LB Leptin receptor signaling in T cells is required for Th17 differentiation. J Immunol (2015) 194(11):5253–60. 10.4049/jimmunol.1402996 PMC4433844 25917102

[62] Yu Y Liu Y Shi F-D Zou H Matarese G La Cava A Cutting Edge: Leptin-Induced RORγt Expression in CD4+ T Cells Promotes Th17 Responses in Systemic Lupus Erythematosus. J Immunol (2013) 190(7):3054–8. 10.4049/jimmunol.1203275 PMC3608794 23447682

[63] Lee GR The Balance of Th17 versus Treg Cells inAutoimmunity. Int J Mol Sci (2018)19:3–17. 10.3390/ijms19030730 PMC5877591 29510522

[64] Dang EV Barbi J Yang HY Jinasena D Yu H Zheng Y Control of T(H)17/T(reg) balance by hypoxia-inducible factor 1. Cell (2011) 146(5):772–84. 10.1016/j.cell.2011.07.033 PMC3387678 21871655

[65] Harjes U Leptin boosts T cell function in tumours. Nat Rev Cancer (2019) 19(11):607. 10.1038/s41568-019-0208-7 31527698

[66] Matarese G Procaccini C De Rosa V Horvath TL La Cava A Regulatory T cells in obesity: the leptin connection. Trends Mol Med (2010) 16(6):247–56. 10.1016/j.molmed.2010.04.002 20493774

[67] De Rosa V Procaccini C Cali G Pirozzi G Fontana S Zappacosta S A key role of leptin in the control of regulatory T cell proliferation. Immunity (2007) 26(2):241–55. 10.1016/j.immuni.2007.01.011 17307705

[68] Wagner N-M Brandhorst G Czepluch F Lankeit M Eberle C Herzberg S Circulating regulatory T cells are reduced in obesity and may identify subjects at increased metabolic and cardiovascular risk. Obesity (2013) 21(3):461–8. 10.1002/oby.20087 23592653

[69] Alwarawrah Y Nichols AG Green WD Eisner W Kiernan K Warren J Targeting T-cell oxidative metabolism to improve influenza survivalin a mouse model of obesity. Int J Obes (2020) 44:2419–29. 10.1038/s41366-020-00692-3 PMC7686301 33037327

[70] Tanaka M Suganami T Kim-Saijo M Toda C Tsuiji M Ochi K Role of Central Leptin Signaling in the Starvation-Induced Alteration of B-Cell Development. J Neurosci (2011) 31(23):8373. 10.1523/JNEUROSCI.6562-10.2011 21653842 PMC6623333

[71] Frasca D Diaz A Romero M Blomberg BB Leptin induces immunosenescence in human B cells. Cell Immunol. (2020) 348:103994. 10.1016/j.cellimm.2019.103994. 31831137 PMC7002206

[72] Agrawal S Gollapudi S Su H Gupta S Leptin Activates Human B Cells to Secrete TNF-α, IL-6, and IL-10 via JAK2/STAT3 and p38MAPK/ERK1/2 Signaling Pathway. J Clin Immunol (2011) 31(3):472–8. 10.1007/s10875-010-9507-1 PMC3132280 21243519

[73] Frasca D Ferracci F Diaz A Romero M Lechner S Blomberg BB Obesity decreases B cell responses in young and elderly individuals. Obesity (2016) 24(3):615–25. 10.1002/oby.21383 PMC4769695 26857091

[74] Mancuso P Gottschalk A Phare SM Peters-Golden M Lukacs NW Huffnagle GB Leptin-deficient mice exhibit impaired host defense in Gram-negative pneumonia. J Immunol (2002) 168(8):4018–24. 10.4049/jimmunol.168.8.4018 11937559

[75] Plotkin BJ Paulson D Chelich A Jurak D Cole J Kasimos J Immune responsiveness in a rat model for type II diabetes (Zucker rat, fa/fa): susceptibility to Candida albicans infection and leucocyte function. J Med Microbiol (1996) 44(4):277–83. 10.1099/00222615-44-4-277 8606356

[76] Altintas MM Nayer B Walford EC Johnson KB Gaidosh G Reiser J Leptin deficiency-induced obesity affects the density of mast cells in abdominal fat depots and lymph nodes in mice. Lipids Health Dis (2012) 11:21. 10.1186/1476-511x-11-21 22313574 PMC3287967

[77] Finlay CM Cunningham KT Doyle B Mills KHG IL-33–Stimulated Murine Mast Cells Polarize AlternativelyActivated Macrophages, Which Suppress T Cells That Mediate Experimental AutoimmuneEncephalomyelitis. J Immunol (2020) 205(7):1909–19. 10.4049/jimmunol.1901321 32859729

[78] Elieh Ali Komi D Shafaghat F Christian M Crosstalk Between Mast Cells and Adipocytes in Physiologic and Pathologic Conditions. Clin Rev Allergy Immunol (2020) 58(3):388–400. 10.1007/s12016-020-08785-7 32215785 PMC7244609

[79] Zarkesh-Esfahani H Pockley AG Wu Z Hellewell PG Weetman AP Ross RJ Leptin indirectly activates human neutrophils via induction of TNF-alpha. J Immunol (2004) 172(3):1809–14. 10.4049/jimmunol.172.3.1809 14734764

[80] Madan R Guo X Naylor C Buonomo EL Mackay D Noor Z Role of leptin-mediated colonic inflammation in defense against Clostridium difficile colitis. Infect Immun (2014) 82(1):341–9. 10.1128/iai.00972-13 PMC3911837 24166957

[81] Kordonowy LL Burg E Lenox CC Gauthier LM Petty JM Antkowiak M Obesity is associated with neutrophil dysfunction and attenuation of murine acute lung injury. Am J Respir Cell Mol Biol (2012) 47(1):120–7. 10.1165/rcmb.2011-0334OC PMC3402797 22427537

[82] Grotta MB Squebola-Cola DM Toro AA Ribeiro MA Mazon SB Ribeiro JD Obesity increases eosinophil activity in asthmatic children and adolescents. BMC Pulm Med (2013) 13:39. 10.1186/1471-2466-13-39 23773659 PMC3686646

[83] Tian Z Sun R Wei H Gao B Impaired natural killer (NK) cell activity in leptin receptor deficient mice: leptin as a critical regulator in NK cell development and activation. Biochem Biophys Res Commun (2002) 298(3):297–302. 10.1016/s0006-291x(02)02462-2 12413939

[84] La Cava A Leptin in inflammation and autoimmunity. Cytokine (2017) 98:51–8. 10.1016/j.cyto.2016.10.011 PMC5453851 27916613

[85] Fernández-Riejos P Najib S Santos-Alvarez J Martín-Romero C Pérez-Pérez A González-Yanes C Role of Leptin in the Activation of Immune Cells. Mediators Inflammation (2010) 2010:568343. 10.1155/2010/568343 PMC2846344 20368778

[86] Banks AS Davis SM Bates SH Myers MG Jr. Activation of downstream signals by the long form of the leptin receptor. J Biol Chem (2000) 275(19):14563–72. 10.1074/jbc.275.19.14563 10799542

[87] MacIver NJ Michalek RD Rathmell JC Metabolic Regulation of T Lymphocytes. Annu Rev Immunol (2013) 31(1):259–83. 10.1146/annurev-immunol-032712-095956 PMC3606674 23298210

[88] Friedline RH Ko HJ Jung DY Lee Y Bortell R Dagdeviren S Genetic ablation of lymphocytes and cytokine signaling in nonobese diabetic mice prevents diet-induced obesity and insulin resistance. FASEB J (2016) 30(3):1328–38. 10.1096/fj.15-280610 PMC4750424 26644351

[89] Patsouris D Li PP Thapar D Chapman J Olefsky JM Neels JG Ablation of CD11c-positive cells normalizes insulin sensitivity in obese insulin resistant animals. Cell Metab (2008) 8(4):301–9. 10.1016/j.cmet.2008.08.015 PMC2630775 18840360

[90] Feng B Jiao P Nie Y Kim T Jun D van Rooijen N Clodronate liposomes improve metabolic profile and reduce visceral adipose macrophage content in diet-induced obese mice. PLoS One (2011) 6(9):e24358–e. 10.1371/journal.pone.0024358 PMC3171445 21931688

[91] Khan IM Dai Perrard XY Perrard JL Mansoori A Smith CW Wu H Attenuated adipose tissue and skeletal muscle inflammation in obese mice with combined CD4+ and CD8+ T cell deficiency. Atherosclerosis (2014) 233(2):419–28. 10.1016/j.atherosclerosis.2014.01.011 PMC4094239 24530773

[92] O’Rourke RW White AE Metcalf MD Winters BR Diggs BS Zhu X Systemic inflammation and insulin sensitivity in obese IFN-gamma knockout mice. Metabol: Clin Exp (2012) 61(8):1152–61. 10.1016/j.metabol.2012.01.018 PMC3457921 22386937

[93] Stolarczyk E Vong CT Perucha E Jackson I Cawthorne MA Wargent ET Improved insulin sensitivity despite increased visceral adiposity in mice deficient for the immune cell transcription factor T-bet. Cell Metab (2013) 17(4):520–33. 10.1016/j.cmet.2013.02.019 PMC3685808 23562076

[94] Matarese G Di Giacomo A Sanna V Lord GM Howard JK Di Tuoro A Requirement for leptin in the induction and progression of autoimmune encephalomyelitis. J Immunol (2001) 166(10):5909–16. 10.4049/jimmunol.166.10.5909 11342605

[95] Siegmund B Lehr HA Fantuzzi G Leptin: a pivotal mediator of intestinal inflammation in mice. Gastroenterology (2002) 122(7):2011–25. 10.1053/gast.2002.33631 12055606

[96] Siegmund B Lear-Kaul KC Faggioni R Fantuzzi G Leptin deficiency, not obesity, protects mice from Con A-induced hepatitis. Eur J Immunol (2002) 32(2):552–60. 10.1002/1521-4141(200202)32:2<552::aid-immu552>3.0.co;2-h 11828372

[97] Tarzi RM Cook HT Jackson I Pusey CD Lord GM Leptin-deficient mice are protected from accelerated nephrotoxic nephritis. Am J Pathol (2004) 164(2):385–90. 10.1016/s0002-9440(10)63128-8 PMC1602275 14742244

[98] De Rosa V Procaccini C La Cava A Chieffi P Nicoletti GF Fontana S Leptin neutralization interferes with pathogenic T cell autoreactivity in autoimmune encephalomyelitis. J Clin Invest (2006) 116(2):447–55. 10.1172/jci26523 PMC1326145 16410832

[99] Sanna V Di Giacomo A La Cava A Lechler RI Fontana S Zappacosta S Leptin surge precedes onset of autoimmune encephalomyelitis and correlates with development of pathogenic T cell responses. J Clin Invest (2003) 111(2):241–50. 10.1172/jci16721 PMC151876 12531880

[100] Batocchi AP Rotondi M Caggiula M Frisullo G Odoardi F Nociti V Leptin as a marker of multiple sclerosis activity in patients treated with interferon-beta. J Neuroimmunol (2003) 139(1-2):150–4. 10.1016/s0165-5728(03)00154-1 12799033

[101] Grunfeld C Zhao C Fuller J Pollack A Moser A Friedman J Endotoxin and cytokines induce expression of leptin, the ob geneproduct. J Clin Invest (1996) 97(9):2152–7. 10.1172/JCI118653 PMC507291 8621806

[102] Landman RE Puder JJ Xiao E Freda PU Ferin M Wardlaw SL Endotoxin stimulates leptin in the human and nonhumanprimate. J Clin Endocrinol Metab (2003) 88(3):1285–91. 10.1210/jc.2002-021393 12629120

[103] Trujillo ME Lee M-J Sullivan S Feng J Schneider SH Greenberg AS Tumor Necrosis Factor α and Glucocorticoid Synergistically Increase Leptin Production in Human Adipose Tissue: Role for p38 Mitogen-Activated Protein Kinase. J Clin Endocrinol Metab (2006) 91(4):1484–90. 10.1210/jc.2005-1901 16403817

[104] Grunfeld C Zhao C Fuller J Pollack A Moser A Friedman J Endotoxin and cytokines induce expression of leptin, the ob gene product, in hamsters. J Clin Invest (1996) 97(9):2152–7. 10.1172/JCI118653 PMC507291 8621806

[105] Sarraf P Frederich RC Turner EM Ma G Jaskowiak NT Rivet DJ,3 Multiple cytokines and acute inflammation raise mouse leptin levels: potential role in inflammatory anorexia. J Exp Med (1997) 185(1):171–5. 10.1084/jem.185.1.171 PMC2196098 8996253

[106] Al-Lahham SA Jaradat N Al-Qub M Hamayel A Assaassa A Hammad F Lipopolysaccharide influence on leptin hormone and tumor necrosis factor-alpha release from human adipose tissue. Eur J Inflamm (2018) 16:2058739218774975. 10.1177/2058739218774975

[107] Maurya R Bhattacharya P Dey R Nakhasi HL Leptin Functions in Infectious Diseases. Front Immunol (2018) 9:2741. 10.3389/fimmu.2018.02741 30534129 PMC6275238

[108] Radigan KA Morales-Nebreda L Soberanes S Nicholson T Nigdelioglu R Cho T Impaired clearance of influenza A virus in obese, leptin receptordeficient mice is independent of leptin signaling in the lung epithelium andmacrophages. PLOS ONE (2014) 9(9):e108138. 10.1371/journal.pone.0108138. 25232724 PMC4169489

[109] Nunziata A Funcke JB Borck G von Schnurbein J Brandt S Lennerz B Functional and Phenotypic Characteristics of Human Leptin Receptor Mutations. J Endocr Soc (2019) 3(1):27–41. 10.1210/js.2018-00123 30560226 PMC6293235

[110] Farooqi IS Matarese G Lord GM Keogh JM Lawrence E Agwu C Beneficial effects of leptin on obesity, T cell hyporesponsiveness, and neuroendocrine/metabolic dysfunction of human congenital leptin deficiency. J Clin Investig (2002) 110(8):1093–103. 10.1172/JCI15693 PMC150795 12393845

[111] Chan JL Matarese G Shetty GK Raciti P Kelesidis I Aufiero D Differential regulation of metabolic, neuroendocrine, and immune function by leptin in humans. Proc Natl Acad Sci U S A (2006) 103(22):8481–6. 10.1073/pnas.0505429103 PMC1482518 16714386

[112] Cheong HS Chang Y Joo EJ Cho A Ryu S Metabolic Obesity Phenotypes and Risk of Cellulitis: A CohortStudy. J Clin Med (2019)8:7–19. 10.3390/jcm8070953 PMC6679047 31262086

[113] Paich HA Sheridan PA Handy J Karlsson EA Schultz-Cherry S Hudgens MG Overweight and obese adult humans have a defective cellular immune response to pandemic H1N1 influenza A virus. Obesity (Silver Spring) (2013) 21(11):2377–86. 10.1002/oby.20383 PMC3695020 23512822

[114] Morgan OW Bramley A Fowlkes A Freedman DS Taylor TH Gargiullo P Morbid obesity as a risk factor for hospitalization and death due to 2009 pandemic influenza A(H1N1) disease. PLoS One (2010) 5(3):e9694. 10.1371/journal.pone.0009694 20300571 PMC2837749

[115] Louie JK Acosta M Samuel MC Schechter R Vugia DJ Harriman K A novel risk factor for a novel virus: obesity and 2009 pandemic influenza A (H1N1). Clin Infect Dis (2011) 52(3):301–12. 10.1093/cid/ciq152 21208911

[116] Falagas ME Kompoti M Obesity and infection. Lancet Infect Dis (2006) 6(7):438–46. 10.1016/S1473-3099(06)70523-0 16790384

[117] Cohen S Danzaki K MacIver NJ Nutritional effects on T-cell immunometabolism. Eur J Immunol (2017) 47(2):225–35. 10.1002/eji.201646423 PMC5342627 28054344

[118] Petrilli CM Jones SA Yang J Rajagopalan H O’Donnell LF Chernyak Y Factors associated with hospitalization and critical illness among 4,103 patients with COVID-19 disease in New York City. medRxiv (2020) 369 m:1966. 10.1101/2020.04.08.20057794 PMC7243801 32444366

[119] Lighter J Phillips M Hochman S Sterling S Johnson D Francois F Obesity in patients younger than 60 years is a risk factor forCovid-19 hospital admission. Clin Infect Dis (2020) 71(15):896–7. 10.1093/cid/ciaa415 PMC7184372 32271368

[120] Kalligeros M Shehadeh F Mylona EK Benitez G Beckwith CG Chan PA Association of Obesity with Disease Severity among Patients withCOVID-19. Obesity (Silver Spring) (2020) 28(7):1200–4. 10.1002/oby.22859 PMC7267224 32352637

[121] Simonnet A Chetboun M Poissy J Raverdy V Noulette J Duhamel A High prevalence of obesity in severe acute respiratory syndrome coronavirus-2 (SARS-CoV-2) requiring invasive mechanical ventilation. Obesity (Silver Spring) (2020). 10.1002/oby.22831 PMC7262326 32271993

[122] Czernichow S Beeker N Rives-Lange C Guerot E Diehl JL Katsahian S Obesity doubles mortality in patients hospitalized for SARS-CoV-2 in Paris hospitals, France: a cohort study on 5795 patients. Obesity (Silver Spring) (2020). 10.1002/oby.23014 PMC7461006 32815621

[123] Popkin BM Du S Green WD Beck MA Algaith T Herbst CH Individuals with obesity and COVID-19: A global perspective on theepidemiology and biological relationships. Obes Rev (2020) 21(11):e13128. 10.1111/obr.13128 32845580 PMC7461480

[124] Alti D Sambamurthy C Kalangi SK Emergence of Leptin in Infection and Immunity: Scope and Challengesin Vaccines Formulation. Front Cell Infect Microbiol(2018) 8:147–165. 10.3389/fcimb.2018.00147 29868503 PMC5954041

